# Effects of the addition of dexamethasone on postoperative analgesia after anterior cruciate ligament reconstruction surgery under quadruple nerve blocks

**DOI:** 10.1186/s12871-021-01440-4

**Published:** 2021-09-08

**Authors:** Yuki Aoyama, Shinichi Sakura, Shoko Abe, Erika Uchimura, Yoji Saito

**Affiliations:** Department of Anesthesiology, Faculty of Medicine, Shimane University, 89-1 Enya-cho, Izumo City, Shimane 693-8501 Japan

**Keywords:** Nerve block, Anesthesia and Analgesia, Pain, Postoperative, Dexamethasone, Anterior cruciate ligament reconstruction

## Abstract

**Background:**

Anterior cruciate ligament (ACL) reconstruction is an invasive surgical procedure for the knee. Quadruple nerve blocks including continuous femoral nerve block and single-injection sciatic, obturator, and lateral femoral cutaneous nerve blocks can provide effective intraoperative anesthesia and analgesia in the early postoperative period. However, severe pain often appears after the effect of single-injection nerve blocks resolves and that is why we conducted two studies. The first study was to determine whether dexamethasone administered along with local anesthetic for sciatic nerve block could prolong the duration of analgesia in patients given quadruple nerve blocks, including continuous femoral nerve block, for ACL reconstruction using a hamstring tendon autograft. The second study was designed to evaluate any difference in effects from dexamethasone administered perineurally versus intravenously.

**Methods:**

Patients undergoing unilateral arthroscopic ACL reconstruction using a hamstring tendon autograft were enrolled into two studies. The first study was prospectively conducted to see if dexamethasone 4 mg could prolong the duration of analgesia when administered perineurally to the subgluteal sciatic nerve with 0.5% ropivacaine. In the second study, we retrospectively evaluated the effects of intravenous dexamethasone 4 mg as compared with those of perineural dexamethasone to the sciatic nerve block and effects with no dexamethasone.

**Results:**

In the first study, perineural dexamethasone prolonged the duration of analgesia by 9.5 h (median duration: 22.5 and 13.0 h with and without perineural dexamethasone, respectively, P = 0.011). In the second study, the duration of analgesia was similarly prolonged for intravenous and perineural dexamethasone compared with no dexamethasone.

**Conclusion:**

Perineural dexamethasone administered along with local anesthetic for single sciatic nerve block prolonged the duration of analgesia of quadruple nerve blocks for ACL reconstruction, however the effects were not different from those of intravenous dexamethasone.

**Trial registration:**

The protocols of both studies were approved by the Institutional Review Board of Shimane University Hospital, Japan (study number 2821 and 3390 for study 1 and study 2, respectively). Study 1 was registered in University Hospital Medical Information Network Clinical Trials Registry (UMIN000028930). Study 2, which was a retrospective study, was not registered.

## Introduction

Since the knee joint and its overlying skin and muscle receive innervation from the lumbar plexus and sciatic nerve, a combination of peripheral nerve blocks may be required for intra- and postoperative pain management in knee surgery. Anterior cruciate ligament (ACL) reconstruction is an invasive surgical operation for the knee and causes long-lasting moderate to severe postoperative pain. Quadruple nerve blocks including continuous femoral nerve block and single-injection subgluteal sciatic, obturator and lateral cutaneous femoral nerve blocks have been shown to provide effective intraoperative anesthesia and postoperative analgesia [1]. However, despite the continuous femoral nerve block, pain becomes severe especially on the posterior aspect of the knee, to which the femoral nerve does not supply innervation, after the effect of the single-injection nerve blocks resolves.

Perineural dexamethasone has been shown to prolong the duration of analgesia after peripheral nerve blocks [2–6]. However, most previous studies have focused on brachial plexus blocks, and little information is available on the effects of a proximal sciatic nerve block. Nor have the effects of dexamethasone in patients receiving multiple peripheral nerve blocks for ACL reconstruction been studied. In addition, the mechanism and site of action has not been elucidated nor has the appropriate route of administration of dexamethasone been determined, i.e., whether dexamethasone should be administered perineurally with local anesthetic or intravenously. Therefore, we conducted a series of two studies to see 1) if dexamethasone 4 mg administered along with local anesthetic for the subgluteal sciatic nerve block could prolong the duration of analgesia after quadruple nerve blocks including continuous femoral nerve block in patients undergoing ACL reconstruction using a hamstring autograft and 2) how the effects of perineural dexamethasone differed from those of dexamethasone administered intravenously. Our hypothesis was that perineural and intravenous dexamethasone prolongs the analgesic duration of quadruple nerve blocks.

## Material and methods

### Study 1

This study was conducted to see if dexamethasone administered with local anesthetic for the subgluteal sciatic nerve block could prolong the analgesic duration and improve postoperative analgesia in patients undergoing ACL reconstruction using a hamstring tendon autograft. This randomized controlled double-blind study was approved by the Shimane University Hospital ethical committee on 28th August, 2017 (study number 2821) and registered in the University Hospital Medical Information Network Clinical Trials Registry (UMIN000028930) on 4th September, 2017. The study was conducted between September 2017 and March 2018. Patients (American Society of Anesthesiologists physical status 1–2) undergoing unilateral arthroscopic ACL reconstruction using an ipsilateral hamstring tendon autograft under quadruple peripheral nerve blocks without spinal anesthesia or general anesthesia were included. Patients who had contraindications to peripheral nerve blocks, a history of diabetes mellitus, or any neurologic disease were excluded. Written informed consent was obtained from 22 patients (and/or a guardian of patients under 20 years old). The patients were randomly divided into two groups to receive 20 ml of 0.5% ropivacaine with dexamethasone sodium phosphate 4 mg (4 mg per 1 ml, Fuji Pharma Co., Ltd., Tokyo, Japan) (group P) or 1 ml of normal saline (group C) for single subgluteal sciatic nerve block in a 1:1 ratio. Our institutional clinical trial center conducted randomization using computer-generated sequence of random numbers and informed an investigator which group the patient would be assigned to on the day before surgery. An anesthesiologist who was not involved in the block procedure, anesthesia or postoperative measurements prepared the local anesthetic solution used for sciatic nerve block without labeling for group allocation. Patients were blinded to their group assignment.

In the operation room, a standard noninvasive monitor was applied and an intravenous line was secured for all patients. Midazolam 1–2 mg and fentanyl 50 μg was intravenously given for sedation before block performance, while the patients remained responsive to verbal cues. All patients received single-injection subgluteal sciatic and obturator nerve blocks, and continuous femoral nerve block under ultrasound guidance. Single-injection lateral cutaneous femoral nerve block was added in patients who did not develop anesthesia on the lateral aspect of the thigh approximately five minutes after the femoral nerve block. The blocks were performed under aseptic technique and the supervision of an experienced regional anesthesiologist (S.S.).

First, subgluteal sciatic nerve block was conducted with a patient in the lateral position with the side to be blocked uppermost. An 1–5 MHz convex transducer (LOGIQ e Premium; GE Healthcare, Tokyo, Japan) was positioned to visualize the short axis view of the sciatic nerve at the subgluteal level, and a 100-mm, 21-gauge block needle (SonoPlex STIM; PAJUNK, Geisingen, Germany) was inserted in-plane under ultrasound image [7]. A nerve stimulator (RasinPlex HRP-10; Hakko, Chikuma, Japan) with a pulse duration of 0.1 ms and a stimulating frequency of 2 Hz was used to confirm the sciatic nerve [8]. The local anesthetic solution according to the study protocol was injected around the target nerve. Then a patient was turned to the supine position to receive femoral, obturator, and lateral cutaneous femoral nerve blocks using a 4–12 MHz linear transducer (LOGIQ e Premium; GE Healthcare, Tokyo, Japan). Femoral nerve block was conducted using a 25-gauge catheter over the needle (Contiplex C; B. Braun, Meisungen, Germany) with short axis view, in-plain approach. The needle was inserted from the lateral to medial direction and 15 ml of 0.5% ropivacaine was injected incrementally around the femoral nerve. The catheter was next placed and fixed with sterile tape. Obturator nerve block was conducted using a 21-gauge block needle (SonoPlex STIM; PAJUNK, Geisingen, Germany), and 10 ml of ropivacaine was injected (5 ml each for two fascial planes; between adductor longus and adductor brevis muscles and between adductor brevis and adductor magnus muscles) [1]. Lateral cutaneous femoral nerve block was added, if necessary, by using 1–3 ml of 1% mepivacaine using a 22-gauge needle under ultrasound guidance [9].

After confirming loss of cold sensation on the knee and lower leg, surgery was started. Patients were sedated as requested with a bolus intravenous injection of midazolam or a continuous infusion of propofol. Fentanyl 50 μg was intravenously injected by the attending anesthesiologist when the surgical anesthesia was deemed inadequate. No additional local anesthetics were administered during surgery. The same orthopedic team conducted each surgery. Continuous infusion of 0.17% levobupivacaine at 4 ml/h and patient control analgesia (PCA) with a bolus of 3 ml (30 min lock out time) using an elastomeric infusion pump (COOPDECH Ballonjector 300 PCA set; Daiken Medical, Izumi, Japan) via the femoral nerve catheter was started immediately after surgery and continued for 48 h. The routine postoperative analgesic regimen consisted of loxoprofen sodium 180 mg/day administered orally. Oral or intravenous acetaminophen and a diclofenac suppository were used for rescue analgesia.

Analgesic duration was assessed by measuring the time from the completion of block procedure to first pain on the knee. Duration of motor block was assessed by measuring the time from block to the first ankle movement. Patients were preoperatively asked to record the times when they first felt noticeable pain on the knee and when they noticed the return of both dorsal flexion and planter flexion of the ankle. Other measurements were conducted by anesthesiologists who were blinded to the group allocation and not involved in the block procedure. Visual analogue pain scores (VAS: 0, no pain; 100, worst pain imaginable) at rest and on movement were assessed at 18, 24 and 48 h after blocks. During that same 48 h period, worst VAS, the number of patient-controlled analgesia (PCA) via the femoral nerve catheter, any additional analgesic required and all complications were also assessed.

### Study 2

Perineural dexamethasone was abandoned in our hospital a few months after the completion of study 1, due to a concern regarding its off-label use in Japan as well as in many other countries and possible mix-up among local anesthetic solutions used for different peripheral nerve blocks. Alternately, dexamethasone 4 mg has been administered intravenously at the discretion of an anesthesiologist since then. Study 2 was conducted retrospectively to evaluate the effects of intravenous dexamethasone 4 mg as compared with those of perineural dexamethasone to the sciatic nerve block and effects with no dexamethasone in patients undergoing ACL reconstruction using a hamstring tendon autograft. The Shimane University Hospital ethical committee gave approval to this study on 15th October, 2018 (study number 3390). We then collected intraoperative and postoperative data of patients who received peripheral nerve blocks as a care standard and were registered in the regional anesthesia database in our department. Written informed consent was waived because the study was limited to pre-existing data. Registry data includes detailed information on block performance, sensory and motor blockade, postoperative pain levels and complications in the early postoperative period (for 48 h). All patients enrolled in study 1 were also included in study 2. Data of patients (ages 14–59 years) undergoing unilateral ACL reconstruction using a hamstring tendon autograft under quadruple nerve blocks as described above without general anesthesia or spinal anesthesia between September 2017 and September 2018 were retrieved. Patients (including those who were enrolled in study 1) were divided into three groups: patients who did not receive dexamethasone (either perineurally or intravenously) (group 1), patients who received dexamethasone 4 mg intravenously (group 2), and patients who received dexamethasone 4 mg along with local anesthetic for subgluteal sciatic nerve block (group 3). Sciatic, femoral and obturator nerve blocks were conducted using 0.5% ropivacaine as described above. The intraoperative anesthesia, postoperative pain management and postoperative assessments were similarly conducted to those in study 1.

### Statistical analysis

The primary outcome of both studies was the duration of analgesia, defined as time after the block procedure until the first pain was felt. The secondary outcomes of the studies included duration of motor block, pain scores and the number of PCA required. In study 1, we hypothesized that perineural dexamethasone 4 mg co-administrated with 0.5% ropivacaine would extend the duration of analgesia compared with 0.5% ropivacaine alone. Sample size calculation for study 1 was conducted based on our preliminary data to detect the 7 h difference with SD of 4.6 h. The results showed that 14 patients (7 in each group) were required (α set at 0.05 and β set at 0.2) and the number was increased to 22 patients (11 in each group) to compensate for potential dropouts. Sample size calculation for study 2 was not conducted because the study was conducted retrospectively.

Statistical analysis was performed using SPSS 23.0 software for Windows (SPSS Inc., Chicago, IL). The two-tailed Student’s t-test was used for parametric statistics and the values were expressed as mean ± SD. The Mann–Whitney U-test and Kruskal–Wallis test were applied for non-parametric statistics and the results were expressed as median (interquartile range). The chi-square test or Fisher’s exact test were used for categorical data. The Bonferroni correction was applied when appropriate and a p-value < 0.05 was considered statistically significant.

## Results

### Study 1

**Fig. 1:**
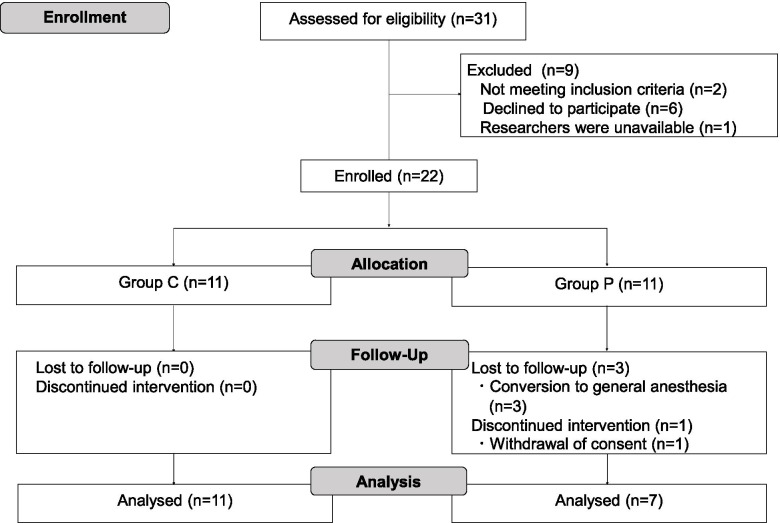
CONSORT 2010 flow diagram for study 1

During the study period, we recruited 22 patients who were randomly allocated into two groups (groups C and P). Four patients in group P were excluded from analysis due to the addition of general anesthesia or withdrawal of consent. The remaining 18 patients completed the study; 11 and 7 patients for groups C and P, respectively (Fig. 1). Patient and intraoperative demographics did not differ between the two groups (Table 1). The duration of analgesia and motor blockade of the sciatic nerve was significantly longer in group P than that in group C (Table 2). No difference was found in VAS scores at rest, on movement or worst pain for 48 h, although patients in group C required a larger number of PCA within 24 h (Table 2). Surgical site infection occurred in one patient in group C. No complications regarding the block were observed.

**Table 1:** Patient demographics and surgical characteristics for study 1

	Group C (n = 11)	Group P (n = 7)	P value
Sex (female), n (%)	5 (45.5%)	4 (57.1%)	1.000
Age, y	28 ± 15	25 ± 11	0.693
Height, cm	164.4 ± 9.5	167.3 ± 15.5	0.624
Body weight, kg	66.8 ± 13.5	62.3 ± 11.7	0.482
BMI, kg/m^2^	24.5 ± 3.1	22.2 ± 2.7	0.131
ASA-PS (1/2), n	6/5	6/1	0.316
Surgical site (right), n (%)	7 (63.6%)	4 (57.1%)	1.000
Procedure with meniscus repair, n (%)	5 (45.5%)	4 (57.1%)	1.000
Surgical time, min	91 ± 23	105 ± 22	0.218
Intraoperative fentanyl, μg	0 (0–50)	0 (0–12.5)	0.659

Data presented as number of patients (%), mean ± standard deviation, or median (interquartile range). BMI, body mass index; ASA-PS, American Society of Anesthesiologists physical status

**Table 2:** Postoperative patient data regarding block duration, pain scores and postoperative analgesic requirements for study 1

	Group C (n = 11)	Group P (n = 7)	P value
Duration of analgesia, h	13.0 (8.5–14.0)	22.5 (15.3–33.4)	0.011
Duration of motor blockade, h	13.0 (13.0–16.0)	20.8 (17.6–25.1)	0.003
VAS at rest, mm			
18 h	38 (30–58)	33 (23–46)	0.425
24 h	21 (18–60)	38 (8–66)	1.000
48 h	12 (0–29)	17 (11–32)	0.246
VAS on movement, mm			
18 h	78 (60–90)	43 (23–67)	0.069
24 h	65 (42–82)	52 (16–76)	0.126
48 h	43 (33–65)	42 (35–68)	0.860
Worst VAS within 48 h, mm	83 (65–100)	74 (54–93)	0.525
PCA, time			
0–24 h	10 (4–15)	3 (0–3)	0.011
24–48 h	3 (0–5)	4 (0–6)	0.536
Rescue analgesics required, time			
0–24 h	3 (1–5)	1 (0–3)	0.085
24–48 h	2 (1–3)	2 (1–2)	0.659

Data presented as median (interquartile range). VAS, visual analogue scale; PCA, patient-controlled analgesia

### Study 2

**Fig. 2:**
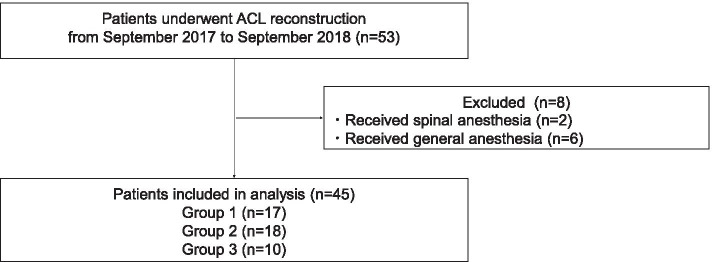
Patient flow diagram for study 2

Forty-five patients were included in the study (17, 18 and 10 patients for groups 1, 2 and 3, respectively) (Fig. 2). The same orthopedic team conducted each surgery. The demographics and surgical characteristics were similar among the three groups of patients (Table 3). The duration of analgesia and motor blockade of sciatic nerve were significantly prolonged in groups 2 and 3 compared with group 1 (Table 4). No difference was observed between groups 2 and 3 in the duration of analgesia and motor blockade. VAS on movement at 18 h and rescue analgesic requirements were significantly higher in group 1 compared with group 2. Patients in group 1 required a significantly larger number of PCA compared with patients in group 3 for 24 h. No significant difference was observed between groups 2 and 3 in either pain scores or analgesic requirements. No severe complications related to blocks were observed.

**Table 3:** Patient demographics and surgical characteristics for study 2

	Group 1 (n = 17)	Group 2 (n = 18)	Group 3 (n = 10)	P value
Sex (female), n (%)	6 (35.3%)	10 (55.6%)	5 (50.0%)	0.472
Age, y	28 ± 15	26 ± 11	23 ± 10	0.662
Height, cm	165.4 ± 8.8	165.4 ± 9.9	166.3 ± 12.9	0.970
Body weight, kg	65.5 ± 11.5	65.6 ± 17.6	62.6 ± 9.6	0.843
BMI, kg/m^2^	23.8 ± 2.8	23.8 ± 4.6	22.6 ± 2.3	0.664
ASA-PS (1/2), n	10/7	13/5	9/1	0.223
Surgical site (right), n (%)	9 (52.9%)	8 (44.4%)	6 (60.0%)	0.719
Procedure with meniscus repair, n (%)	11 (64.7%)	5 (27.8%)	4 (40.0%)	0.085
Surgical time, min	88 (75–102)	81 (73–83)	106 (84–129)	0.489
Intraoperative fentanyl, μg	0 (0–25)	0 (0)	0 (0)	0.677

Data presented as number of patients (%), mean ± standard deviation, or median (interquartile range). BMI, body mass index; ASA-PS, American Society of Anesthesiologists physical status

**Table 4:** Postoperative patient data regarding block duration, pain scores and postoperative analgesic requirements for study 2

	Group 1 (n = 17)	Group 2 (n = 18)	Group 3 (n = 10)	P value
Duration of analgesia, h	13.0 (8.5–14.0)	20.0 (16.8–22.5)*	22.5 (15.3–33.4)*	< 0.0001
Duration of motor blockade, h	13.0 (10.0–15.5)	17.0 (9.0–21.0)*	20.8 (17.6–25.1)*	< 0.0001
VAS at rest, mm				
18 h	36 (21–56)	26 (3–43)	33 (23–46)	0.051
24 h	21 (18–59)	29 (13–70)	38 (8–66)	0.546
48 h	14 (0–27)	20 (6–29)	17 (11–32)	0.389
VAS on movement, mm				
18 h	73 (60–90)	29 (3–49)*	43 (23–67)	0.003
24 h	65 (44–82)	40 (17–70)	52 (16–76)	0.042
48 h	52 (34–64)	80 (35–80)	42 (35–68)	0.077
Worst VAS within 48 h, mm	82 (62–100)	80 (55–82)	74 (54–93)	0.358
PCA, time				
0–24 h	10 (6–14)	2 (1–10)	3 (0–3)*	0.004
24–48 h	4 (0–5)	6 (0–13)	4 (0–6)	0.429
Rescue analgesics required, time				
0–24 h	3 (1–5)	1 (0–3)*	1 (0–3)	0.028
24–48 h	2 (1–4)	2 (1–3)	2 (1–2)	0.382

Data presented as median (interquartile range). *P < 0.005 vs group 1. VAS, visual analogue scale; PCA, patient-controlled analgesia

## Discussion

We conducted a series of two studies. In the first study, we conducted a prospective randomized controlled double-blind study to see if dexamethasone administered with local anesthetic to the subgluteal approach to sciatic nerve block prolonged the duration of analgesia provided with quadruple nerve blocks in patients undergoing ACL reconstruction using a hamstring tendon graft. Then the second study was conducted to see how the effects differed between different routes for dexamethasone administration, i.e., perineural and intravenous administration. Since comparative studies involving off-label drug usage have become difficult to conduct since the implementation of the Clinical Trials Act in April, 2018 in Japan, study 2 was retrospectively conducted by simply using data stored in our registry. In these two studies, we found that dexamethasone effectively prolonged the duration of postoperative analgesia with quadruple nerve blocks both when administered perineurally and intravenously for subgluteal sciatic nerve block. We also found that the administration of perineural and intravenous dexamethasone similarly prolonged the duration of analgesia by 7–10 h. These extra hours of analgesia should have greatly benefited patients by allowing most of them to spend the night after surgery without pain.

To the best of our knowledge, this is the first study evaluating the effects of dexamethasone as an adjuvant to proximal sciatic nerve block for ACL reconstruction. Most of the previous studies evaluating the effects of the addition of dexamethasone have been conducted with upper limb blocks. The present results confirm that the addition of dexamethasone also prolongs the duration of analgesia and motor block of proximal lower extremity blocks. Rahangdale et al. studied patients receiving an ultrasound-guided infragluteal-parabiceps approach (similar to our approach) to sciatic nerve block using 0.5% bupivacaine and epinephrine for ankle and foot surgery. They found that the addition of dexamethasone 8 mg prolonged the analgesia by mean duration of 13 h compared with normal saline control [10].

Although the optimal dose of dexamethasone as an adjuvant for peripheral nerve blocks is still inconclusive, the 4 mg dose of dexamethasone was used in the present study for the following reasons. To minimize adverse effects, a drug should be administered in a minimal amount that can still produce the highest efficacy possible. Recent studies have explored the effects of lower doses of dexamethasone and have found that even a dose as small as 1 mg can effectively prolong the duration of analgesia [11]. Albrecht et al. showed that 1–4 mg of perineural dexamethasone extended the duration of analgesia for interscalene block dose-dependently [12]. In addition, a previous meta-analysis showed a ceiling dose of 4 mg for perineural administration [13]. Therefore, we considered dexamethasone 4 mg as the most effective minimum dose.

To date, the mechanism by which dexamethasone exerts the effects on extending the duration of analgesia is still unclear. Results of previous studies have suggested that dexamethasone acts both locally and systemically. Possible mechanisms include a direct inhibition of signal transmission in nociceptive C-fibers [14], up-regulation of potassium channels [15], locally induced vasoconstriction [16, 17], and systemic anti-inflammatory effects [18]. Previous meta-analyses have shown that the addition of dexamethasone along with local anesthetics extends analgesia after peripheral nerve blocks by 6–10 h [2–6] and that the extension is longer with perineural administration compared with intravenous administration by 3–4 h [6, 19–22]. However, most of these results were obtained from studies using brachial plexus blocks, and some studies using lower extremity blocks have shown different results. Fredrickson, et al. conducted a comparative study between systemic and perineural dexamethasone for sciatic nerve block in patients undergoing ankle surgery [23]. They reported that perineural dexamethasone 8 mg had only a minor analgesic enhancing effect compared with systemic administration. Jæger et al. conducted a volunteer study and found that the duration of bilateral saphenous nerve blocks with or without dexamethasone were not different from each other [24]. The results suggest that perineurally administered dexamethasone was absorbed and acted systemically to prolong the duration of another block. In the present study, we conducted multiple peripheral nerve blocks and administered dexamethasone perineurally only for sciatic nerve block hoping to see if the dexamethasone also acted as an adjuvant for the other blocks. The number of PCA used for femoral nerve block was smaller in patients receiving perineural dexamethasone compared with control in both studies. Therefore, although no difference was observed in pain scores, it is possible that perineural dexamethasone administered to sciatic nerve was also effective to femoral nerve block.

The present studies have several limitations. First, the second study was conducted retrospectively and included patients also enrolled in the first study. This is mainly because perineural administration of dexamethasone is off-label. Recently, prospective randomized clinical studies including off-label drug usage have become extremely difficult to conduct in Japan and we also stopped administering dexamethasone perineurally in our daily clinical practice soon after the completion of the first study. Eventually, study 2 included a limited number of new patients given perineural dexamethasone and there is a significant difference in time of surgery between patients in groups 2 and 3. Therefore, although intravenous dexamethasone was conducted by discretion of the attending anesthesiologists not participating in the data collection or analysis and no statistical difference was observed in patient demographics between the three groups, it is possible that there was a selection bias. Second, power analysis was not conducted for study 2, therefore the study might be underpower to detect the difference between intravenous and perineural dexamethasone. Thus, it is possible there is little, if any, difference between the two. Third, the duration of analgesia and motor blockade was assessed by asking patients when they felt the analgesia and motor blockade was resolved. Fourth, we conducted obturator nerve block, of which no assessment was made for the block efficacy because sensory innervation of the obturator nerve is quite variable. Finally, since quadruple nerve blocks conducted in the present studies included continuous femoral nerve block, the effects of adding dexamethasone on the blockade of femoral nerve that is the primary sensory nerve for the postoperative pain after ACL reconstruction were not technically assessed. We might have observed different results with single femoral nerve block.

## Conclusion

In conclusion, perineural dexamethasone administered with local anesthetic for single sciatic nerve block prolonged the analgesic duration of quadruple nerve blocks for ACL reconstruction using a hamstring tendon graft, however the effects were not different from those of intravenous dexamethasone.

## Data Availability

The analyzed data sets generated during the study are available from the corresponding author on reasonable request.

## Abbreviations

ACL: Anterior cruciate ligament
PCA: Patient-controlled analgesia
VAS: Visual analogue scale
BMI: Body mass index
ASA-PS: American Society of Anesthesiologists physical status
